# Charge reversible calcium phosphate lipid hybrid nanoparticle for siRNA delivery

**DOI:** 10.18632/oncotarget.17484

**Published:** 2017-04-27

**Authors:** Rong-Qiao Cai, Dao-Zhou Liu, Han Cui, Ying Cheng, Miao Liu, Bang-Le Zhang, Qi-Bing Mei, Si-Yuan Zhou

**Affiliations:** ^1^ Department of Pharmaceutics, School of Pharmacy, Fourth Military Medical University, Xi’an, 710032, China; ^2^ Key Laboratory of Gastrointestinal Pharmacology of Chinese Materia Medica of the State Administration of Traditional Chinese Medicine, Fourth Military Medical University, Xi’an, 710032, China

**Keywords:** lipid hybrid nanoparticles, charge conversional, calcium phosphate, siBcl-2

## Abstract

Bcl-2 gene is an important target to treat lung cancer. The small interference RNA (siRNA) of Bcl-2 gene (siBcl-2) can specifically silence Bcl-2 gene. However, naked siBcl-2 is difficult to accumulate in the tumor tissue to exert its activity. In this paper, a calcium phosphate lipid hybrid nanoparticle that possessed charge reversible property was prepared to enhance the activity of siBcl-2 *in vivo*. The average diameter and zeta potential of siBcl-2 loaded calcium phosphate lipid hybrid nanoparticles (LNPS@siBcl-2) were 80 nm and −13 mV at pH7.4 whereas the diameter and zeta potential changed to 1506 nm and +9 mV at pH5.0. LNPS@siBcl-2 could efficiently deliver siBcl-2 to the cytoplasm and significantly decreased the expression of Bcl-2 in A549 cells. Moreover, the *in vivo* experimental results showed that most of the Cy5-siBcl-2 accumulated in tumor tissue after LNPS@Cy5-siBcl-2 was administered to tumor-bearing mice by tail vein injection. Meanwhile, the expression of Bcl-2 was decreased but the expression of the BAX and Caspase-3 was increased in tumor tissue. LNPS@siBcl-2 significantly inhibited the growth of tumor in tumor-bearing mice without any obvious systemic toxicity. Thus, the charge reversible calcium phosphate lipid hybrid nanoparticle was an excellent siBcl-2 delivery carrier to improve the activity of siBcl-2 *in vivo*. LNPS@siBcl-2 has potential in the treatment of lung cancer.

## INTRODUCTION

In 2012, there were 1.82 million new lung cancer cases in the world. 1.56 million deaths were resulted from lung cancer, which took up 19.4% of death from cancer. Lung cancer is the one with the highest morbidity and mortality rate in China. Bcl-2 gene is an important target to treat lung cancer, and the growth of lung cancer can be inhibited by silencing Bcl-2 gene in tumor cells [1–3]. At present, RNA interference (RNAi) therapy has arisen as a new technique for anticancer therapy owning to its specific gene silencing effect [4, 5]. To construct an effective delivery carrier for small interference RNA (siRNA) is the key step for RNAi therapy [6, 7]. Up to now, the siRNA delivery carrier included virus-based vehicle, lipid-based nanoparticles, [8, 9] atelocollagen nanoparticles [10] and polyethyleneimine [11] etc. However, the *in vivo* transfection efficiency and safety of those carriers need to be further improved.

Calcium phosphate, a kind of non-viral siRNA vector, has been broadly used in gene transfect on cells for 40 years because of its inappreciable toxicity, simplicity of production and noticeable transfection efficiency [12–14]. But naked calcium phosphate-nucleic acids particles tend to grow promptly, which results in poor repeatability *in vitro* to limit its use *in vivo* [15]. Hence, to prepare nanosized and colloidal stable calcium phosphate nanoparticles in which nucleic acids are condensed to avoid to be degraded in blood circulation is of great importance [16].

To improve the stability of siRNA loaded calcium phosphate nanoparticles, the nanoparticle should contain a water soluble shell, which is stable in blood stream but microenvironment-sensitive in the targeting tissue by responding to local stimuli such as pH [17]. In addition, after siRNA loaded calcium phosphate nanoparticles are uptaken by the cells, they usually localize in the endolysosome, which leads to the significant degradation of siRNA, and subsequently reducing the transfection efficiency. Thus, an ideal siRNA loaded calcium phosphate nanoparticle should have negative charged surface in systemic circulation but can reverse to a cationic one in acidic endolysosome environment, which is supposed to have the characteristics of long circulation time in blood stream and endolysosomal escape [18, 19]. Based on above hypothesis, the charge-reversible strategy is applied to improving the siRNA delivery efficiency by enhancing endolysosome escape capacity [20–23].

**Figure 1:**
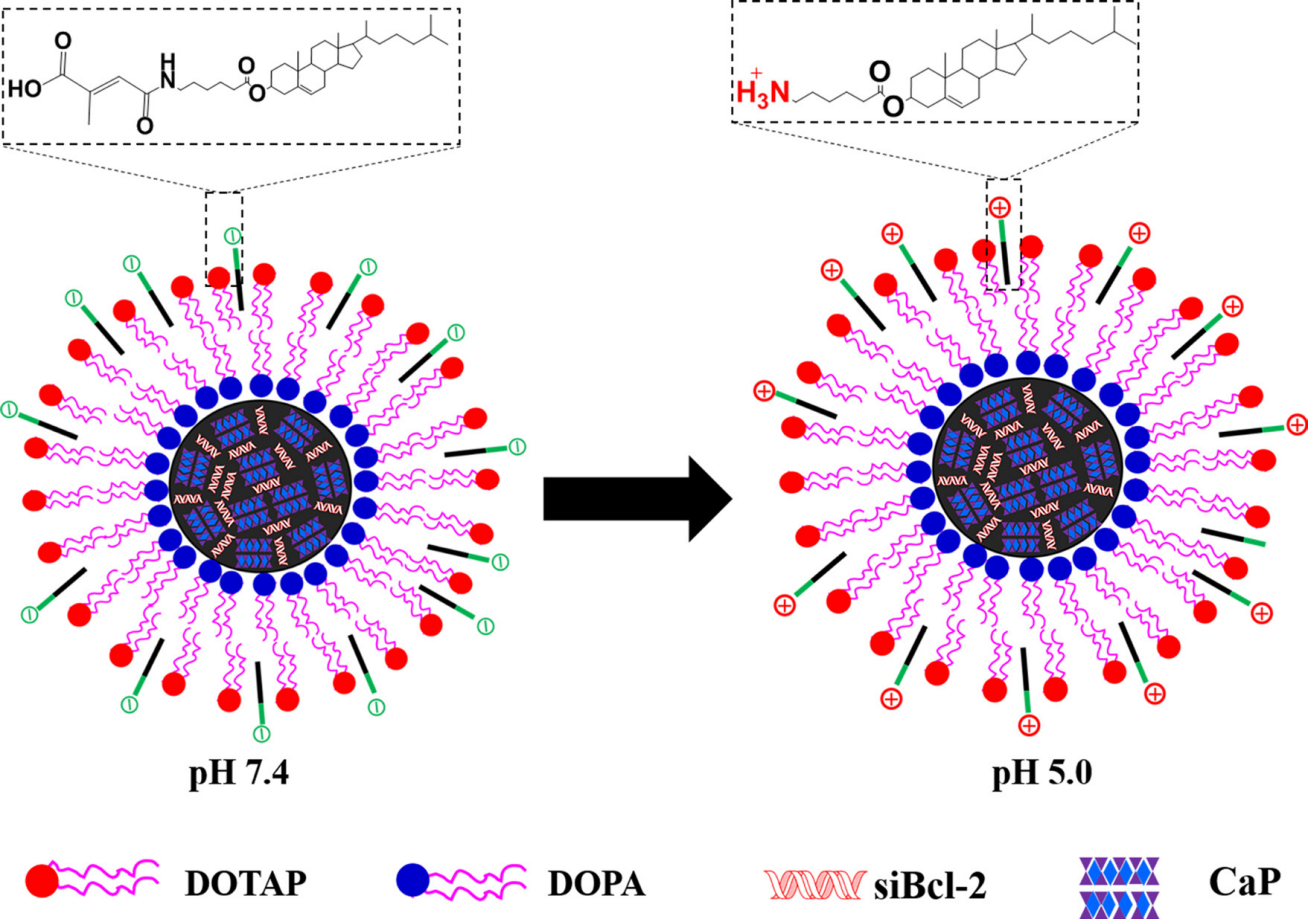
Schematic representation of LNPS@siBcl-2

It has been reported that maleic amide derivatives such as cis-aconitic amide and citraconic amide are sensitive to pH. They possess negative charge at neutral pH but degrade rapidly at acidic environment to expose positively charged amines groups [24, 25] In this study, a pH-triggered charge reversible cholesterol-aminocaproicacid-citraconic (CHOL-AA-Cit) conjugate was synthesized and coated on the surface of siRNA (siBcl-2) loaded calcium phosphate nanoparticles. As shown in Figure 1, when siBcl-2 loaded nanoparticles are uptaken in the acidic intracellular organelles such as endolysosome, CHOL-AA-Cit can rapidly release Cit to form quaternary ammonium, and the surface charge of nanoparticle becomes positive. The charge reversible characteristics can facilitate the escape of nanoparticles from endolysosome through disrupting endolysosomal membrane, subsequently enhancing the release of siRNA to cytoplasm and degrade the target mRNA.

## RESULTS

### Characterization of CHOL-AA-Cit

^1^H NMR (CD_3_Cl) spectrum of CHOL-AA-Cit is shown in Supplementary Figure 1A, δ = 7.99 (1H, d, -CONH-, *J* = 0.8 HZ), 3.33–3.41 (2H, m,-NH-CH_2_-), 2.30 (3H, d, -CH_3_-C = C-, *J* = 4.0 HZ), 2.14–1.08 (46H, m, aliphatic group), 1.00 (3H, d, -CH_3_-, *J* = 3.6 HZ), 0.85 (6H, d, -CH_3_-CH_2_-CH_3_-, *J* = 6.4 HZ), 0.667 (3H, d, -CH_3_-, *J* = 2.8 HZ), 0.90–0.91 (3H, m, -CH_3_-). Mass spectrum of CHOL-AA-Cit is shown in Supplementary Figure 1B, [M-H]^−^ = 612.

### Characterization of LNPS@siBcl-2

**Figure 2:**
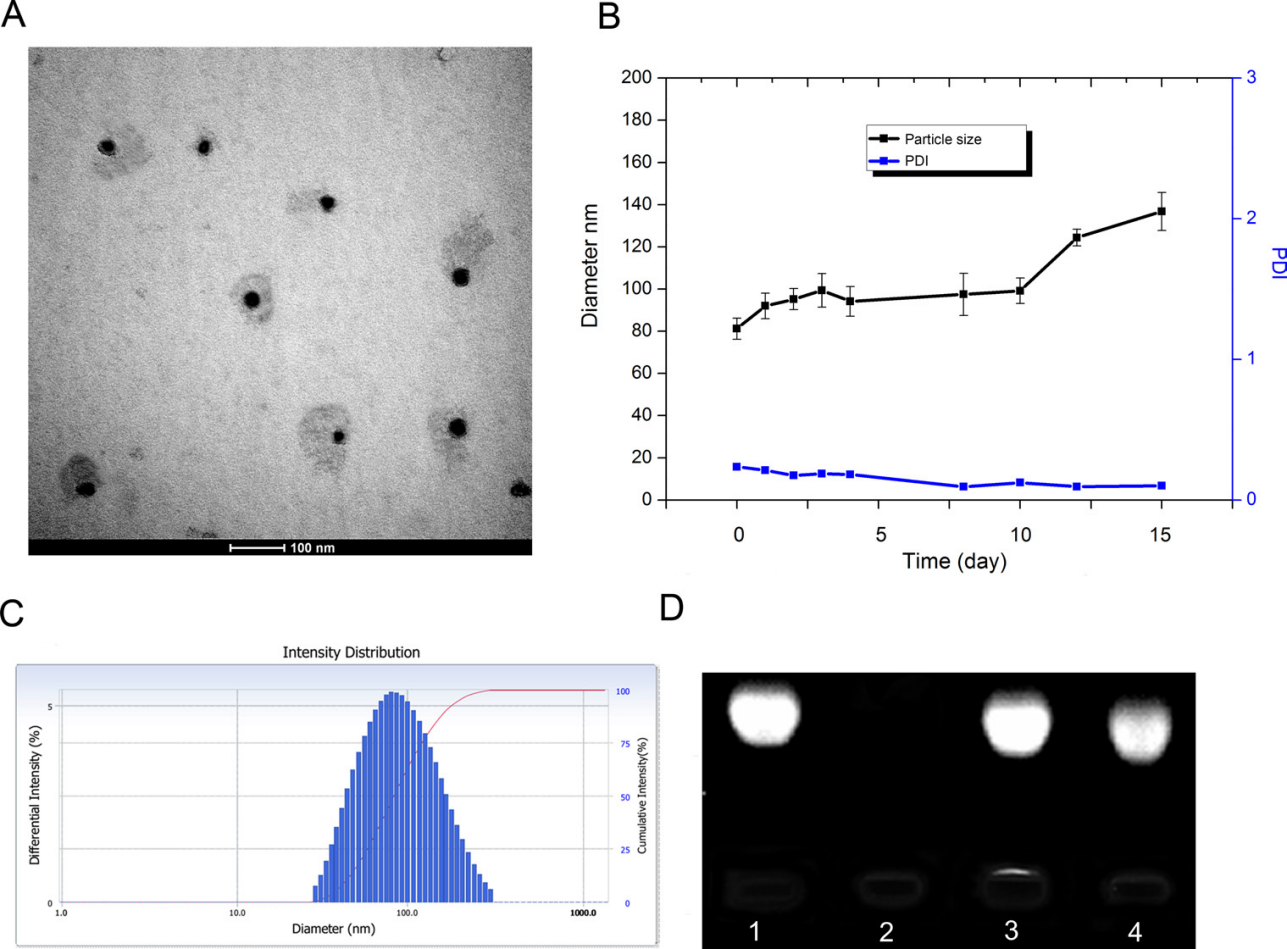
Characteristics of LNPS@siBcl-2 (Panel **A**) is TEM images of LNPS@siBcl-2. (Panel **B**) is the stability of LNPS@siBcl-2 in PBS pH7.4 solution. (Panel **C**) is particle size distribution of LNPS@siBcl-2. (Panel **D**) is the stability of siBcl-2 in LNPS@siBcl-2 against RNase A digestion, following incubation at 37°C for 3 h. Lane 1: free siBcl-2, lane 2: free siBcl-2+RNase A, lane 3: LNPS@siBcl-2, lane 4: LNPS@siBcl-2+RNase A. Data are expressed as the mean ± SD (*n* = 3).

When LNPS@siBcl-2 was prepared, the optimized ratio of CHOL-AA/DOTAP/CHOL-AA-Cit was 150/225/150 (μL/μL/μL), and the concentration of lipid was 10 mmol/L. As shown in Figure 2, the hybrid nanoparticles exhibited relatively spherical shape. The average particle size of LNPS@siBcl-2 was 80 nm in PBS (pH7.4). LNPS@siBcl-2 was stable in 10 days in PBS (pH7.4). The siBcl-2 encapsulated in hybrid nanoparticles was stable when LNPS@siBcl-2 incubated with RNase A for 2 h (Figure 2D). This result implied LNPS@siBcl-2 protected siBcl-2 from RNase A degradation.

**Figure 3:**
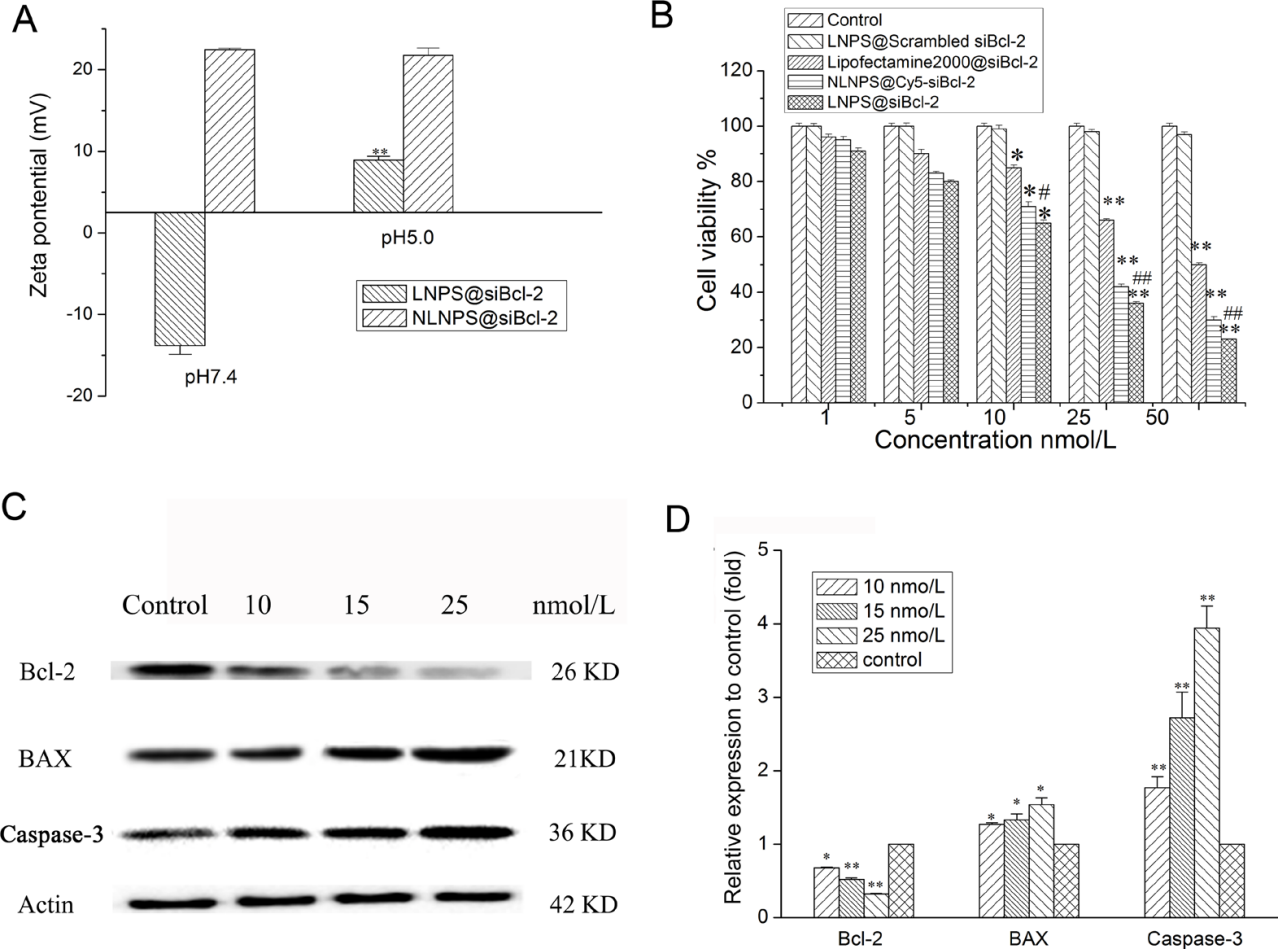
Effect of the medium pH on the zeta potential of LNPS@siBcl-2 (panel **A**), and gene silencing effect of LNPS@siBcl-2 on A549 cells. (Panel **B**) is the cytotoxicity of LNPS@siBcl-2 on A549 cells. (Panel **C**) is the Western Blot analysis of Bcl-2, BAX and Caspase-3 on A549 cells. (Panel **D**) is the statistic result of protein expression. Data are expressed as the mean ± SD, *n* = 3. *p < 0.05, **p < 0.01, vs control, ^#^*p* < 0.05, vs Lipofactemine2000@siBcl-2.

As shown in Figure 3A, the zeta potential of LNPS@siBcl-2 was -13 mV in pH 7.4 medium. Nevertheless, the zeta potential of LNPS@siBcl-2 was +9 mV and average particle size of LNPS@siBcl-2 was approximately 1600 nm in pH 5.5 medium (Supplementary Figure 2). In addition, compared with LNPS@siBcl-2, the zeta potential and average particle size of NLNPS@siBcl-2 (without CHOL-AA-Cit) exhibited no significant changes in pH 7.4 medium and in pH 5.5 medium. As shown in Table 1, LNPS@siBcl-2 significantly destroyed the erythrocyte and caused obvious hemolysis in pH 5.0 medium as compared with what it did in pH 7.4 medium. The above results indicated that the charge-reversal nanoparticles were successfully prepared.

**Table 1:** The hemolysis rate of LNPS@siBcl-2 in different PBS buffer

pH	Hemolysis rate of LNPS@siBcl-2 (%)		
	25 (μg/mL)	50 (μg/mL)	100 (μg/mL)
7.4	0.10 ± 0.05	1 ± 0.5	2 ± 1
5.0	10 ± 2**	67 ± 6**	99 ± 10**

**p < 0.01, vs pH 7.4 at the same concentration.

### Gene silencing effect of LNPS@siBcl-2

The cytotoxicity of LNPS@siBcl-2 on A549 cells is shown in Figure 3B. LNPS@siBcl-2 exhibited higher cytotoxicity as compared with Lipofectamine2000@siBcl-2 at the same concentration of siBcl-2. LNPS@Scrambled Bcl-2 exhibited no cytotoxicity on A549 cells. In addition, NLNPS@siBcl-2 (without AA-Cit conjugate) exhibited lower cytotoxicity on A549 cells than what LNPS@siBcl-2 did. As shown in Figure 3C–3D, LNPS@siBcl-2 significantly decreased the protein expression of Bcl-2 in A549 cells in concentration-dependent manner. In addition, LNPS@siBcl-2 obviously increased the protein expression of BAX and Caspase-3 in A549 cells in concentration-dependent manner.

The calcium phosphate lipid hybrid nanoparticles without loading siBcl-2 (namely LNPS) exhibited almost none cytotoxicity on A549 cells (Supplementary Figure 3) when the concentration of calcium phosphate lipid hybrid nanoparticles ranged from 5 μg /mL to 100 μg/mL.

### Cellular uptake of LNPS@Cy5-siBcl-2 on A549 cells

**Figure 4:**
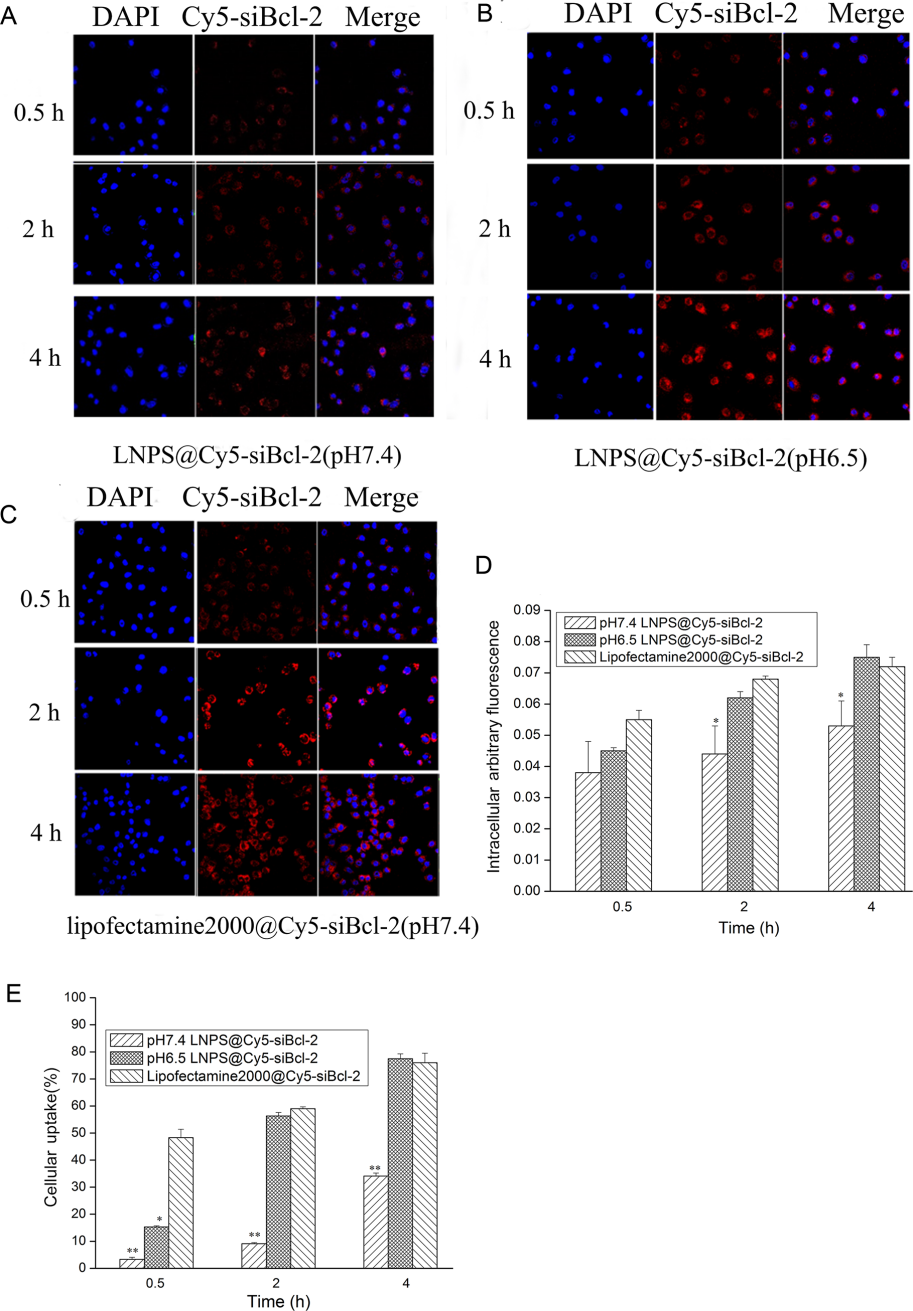
The cellular uptake of LNPS@Cy5-siBcl-2 and Lipofectamine2000@Cy5-siBcl-2 on A549 cells (Panel **A**) and (Panel **B)** are the cellular uptake of LNPS@Cy5-siBcl-2 detected by CLMS in pH7.4 and pH6.5 medium, respectively. (Panel **C**) is the cellular uptake of Lipofectamine2000@Cy5-siBcl-2 detected by CLMS in pH7.4 medium. (Panel **D**) is the statistic result of panel A, panel B and panel C. (Panel **E)** is the cellular uptake of LNPS@Cy5-siBcl-2 and Lipofectamine2000@Cy5-siBcl-2 detected by flow cytometry. 20×oil immersion objective and 10×ocular lens. Red stands for Cy5-siBcl-2, and blue stands for nucleus. Data are expressed as the mean ± SD, *n* = 3. *p < 0.05, **p < 0.01, *vs* Lipofactemine2000@Cy5-siBcl-2 at the same Cy5-siBcl-2 concentration.

The cellular uptake of LNPS@Cy5-siBcl-2 and Lipofectamine2000@Cy5-siBcl-2 on A549 cells was investigated by laser confocal microscopy (LCMS), and the results are shown in Figure 4A–4D. The red fluorescence of Cy5-siBcl-2 could be observed in A549 cells after A549 cells were incubated with LNPS@Cy5-siBcl-2 or Lipofectamine2000@Cy5-siBcl-2 for 0.5 h. The red fluorescence of Cy5-siBcl-2 in the A549 cells increased in time-dependent manner. In addition, cellular uptake of LNPS@Cy5-siBcl-2 in A549 cells in pH 6.5 medium was faster and greater than that in pH 7.4 medium. The cellular uptake of LNPS@Cy5-siBcl-2 and Lipofectamine2000@Cy5-siBcl-2 on A549 cells were also investigated by flow cytometry, the results were consistent with those of LCMS experiment (Figure 4E).

**Figure 5:**
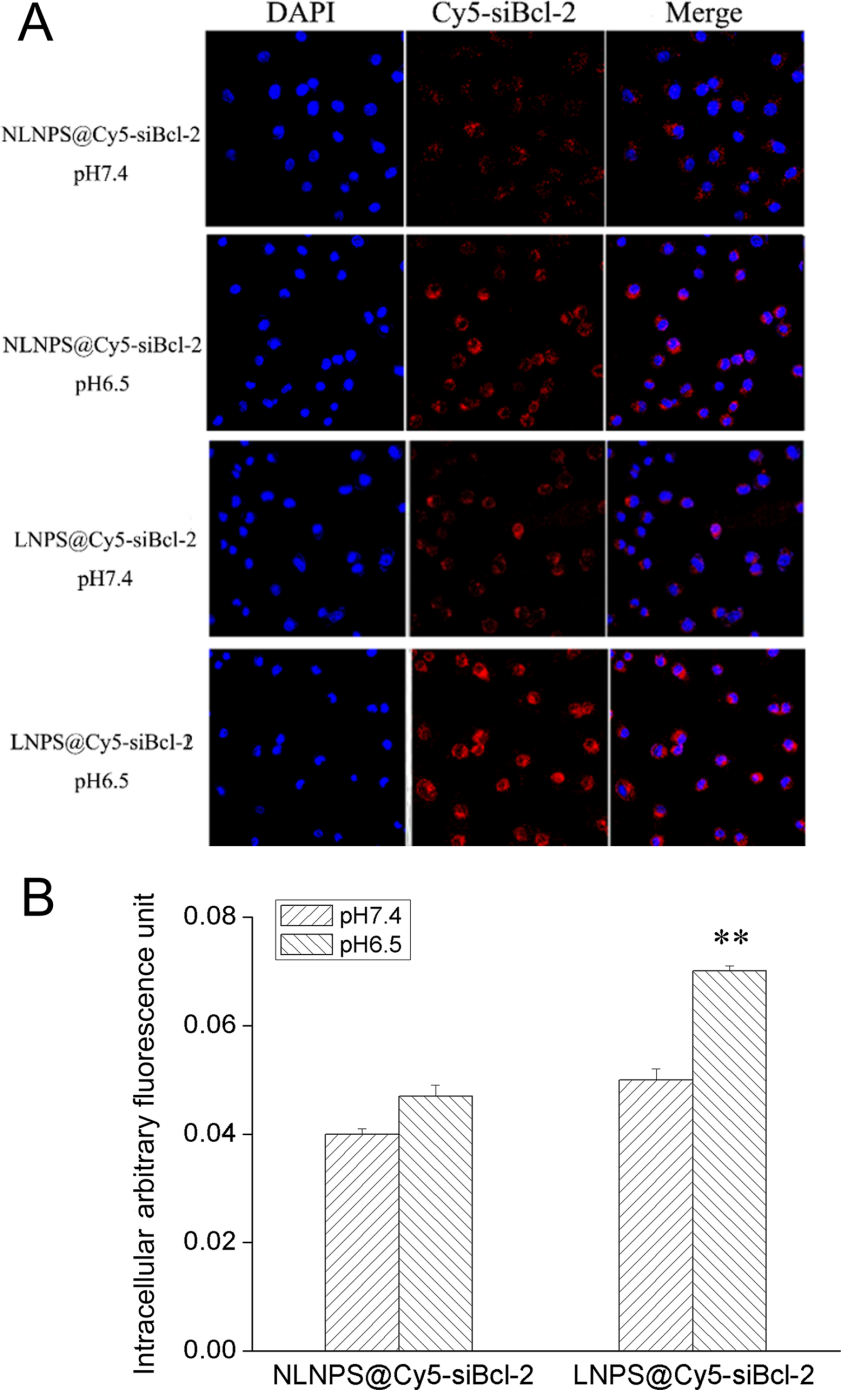
The cellular uptake of LNPS@Cy5-siBcl-2 and NLNPS@Cy5-siBcl-2 on A549 cells in 4 h (Panel **A)** are the cellular uptake of LNPS@Cy5-siBcl-2 and NLNPS@Cy5-siBcl-2 detected by CLMS in pH7.4 and pH6.5 culture medium. (Panel **B**) is the statistic result of panel A. 20 × oil immersion objective and 10 × ocular lens. Red indicates Cy5-siBcl-2, and blue indicates nucleus. Data are expressed as the mean ± SD, *n* = 3. **p < 0.01, vs NLNPS@Cy5-siBcl-2 in the same pH value.

Furthermore, the cellular uptake of LNPS@siBcl-2 and NLNPS@siBcl-2 (without AA-Cit conjugate) on A549 cells were investigated, and the results were shown in Figure 5. The results indicated that there were not significant difference in cellular uptake between LNPS@siBcl-2 and NLNPS@siBcl-2 on A549 cells in pH7.4 culture medium. However, compared with NLNPS@siBcl-2, the cellular uptake of LNPS@siBcl-2 on A549 cells was significantly enhanced in pH6.5 culture medium. The above results implied that the modification of AA-Cit conjugate on calcium phosphate lipid nanoparticle could increase the cellular uptake of calcium phosphate lipid nanoparticle in acidic tumor microenvironment.

**Figure 6:**
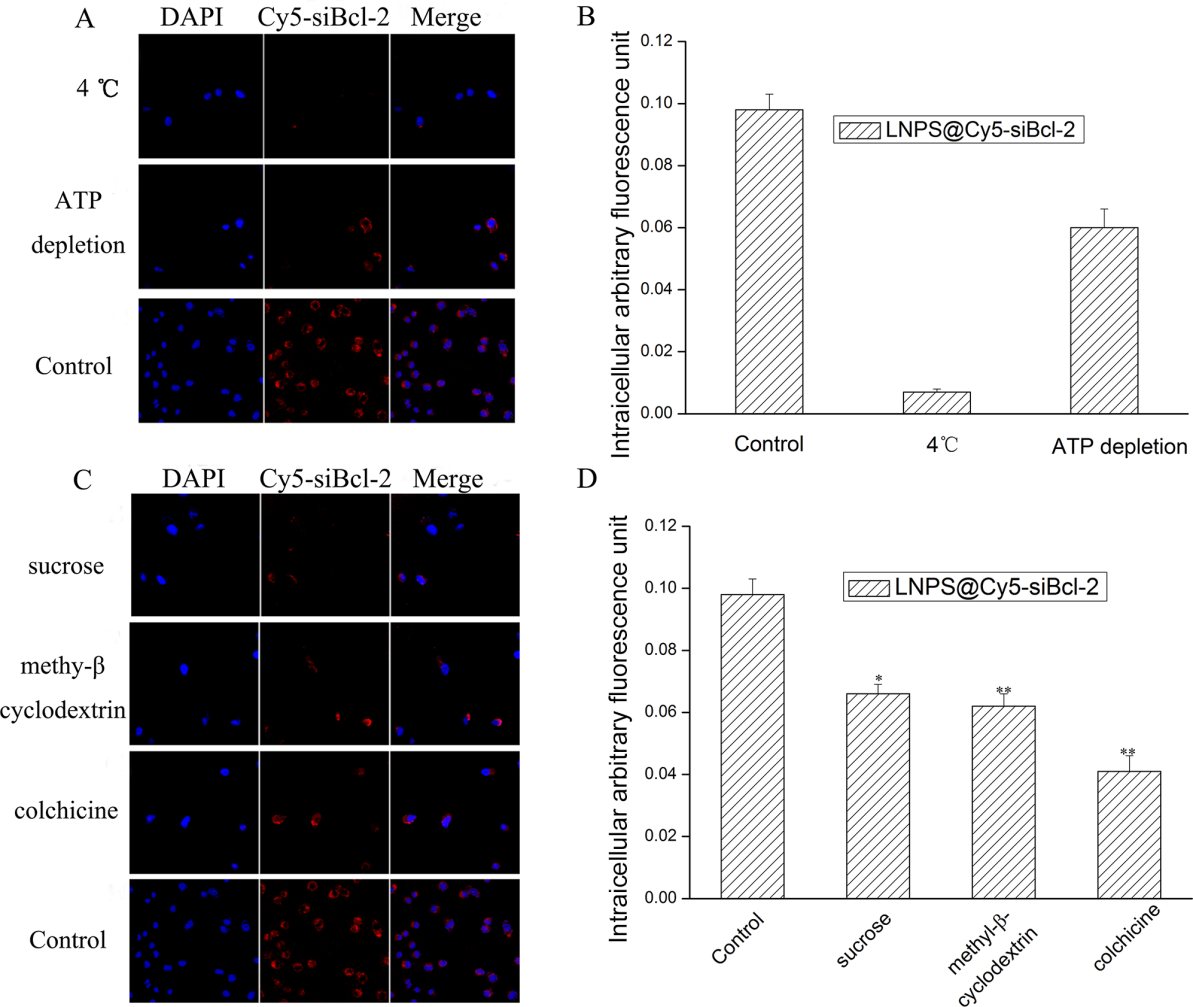
The mechanism of cellular uptake of LNPS@Cy5-siBcl-2 in A549 cells (Panel **A**) is the effect of low temperature (4°C) and ATP depletion on cellular uptake. (Panel **B**) is statistic result of panel A. (Panel **C**) is the effect of endocytic inhibitors on cellular uptake of LNPS@Cy5-siBcl-2. (Panel **D**) is the statistic result of Panel C. 20 × oil immersion objective and 10 × ocular lens. Red stands for Cy5-siBcl-2, and blue stands for nucleus. Data are expressed as the mean ± SD, *n* = 3. *p < 0.05, **p < 0.01, *vs* control.

The mechanism of cellular uptake of LNPS@Cy5-siBcl-2 was observed by laser confocal scan microscopy and flow cytometry, and the results are shown in Figure 6. The low temperature (4°C) and ATP depletion decreased the cellular uptake of the LNPS@Cy5-siBcl-2, which indicated the endocytosis of LNPS@Cy5-siBcl-2 was an energy-consuming process. Meanwhile, the cellular uptake of LNPS@Cy5-siBcl-2 was 42% of control at the present of colchicine, indicating that the micropinocytosis was the main pathway for the endocytosis of LNPS@Cy5-siBcl-2 on A549 cells. Besides, the cellular uptake of LNPS@Cy5-siBcl-2 became 68% and 63% of control at the present of sucrose and methyl-β-cyclodextrin, respectively. This indicated that clathrin-associated and the caveolae-mediated procedures were also involved in the endocytosis of LNPS@Cy5-siBcl-2.

### Intracellular trafficking of Cy5-siBcl-2 delivered by LNPS@Cy5-siBcl-2 in A549 cells

**Figure 7:**
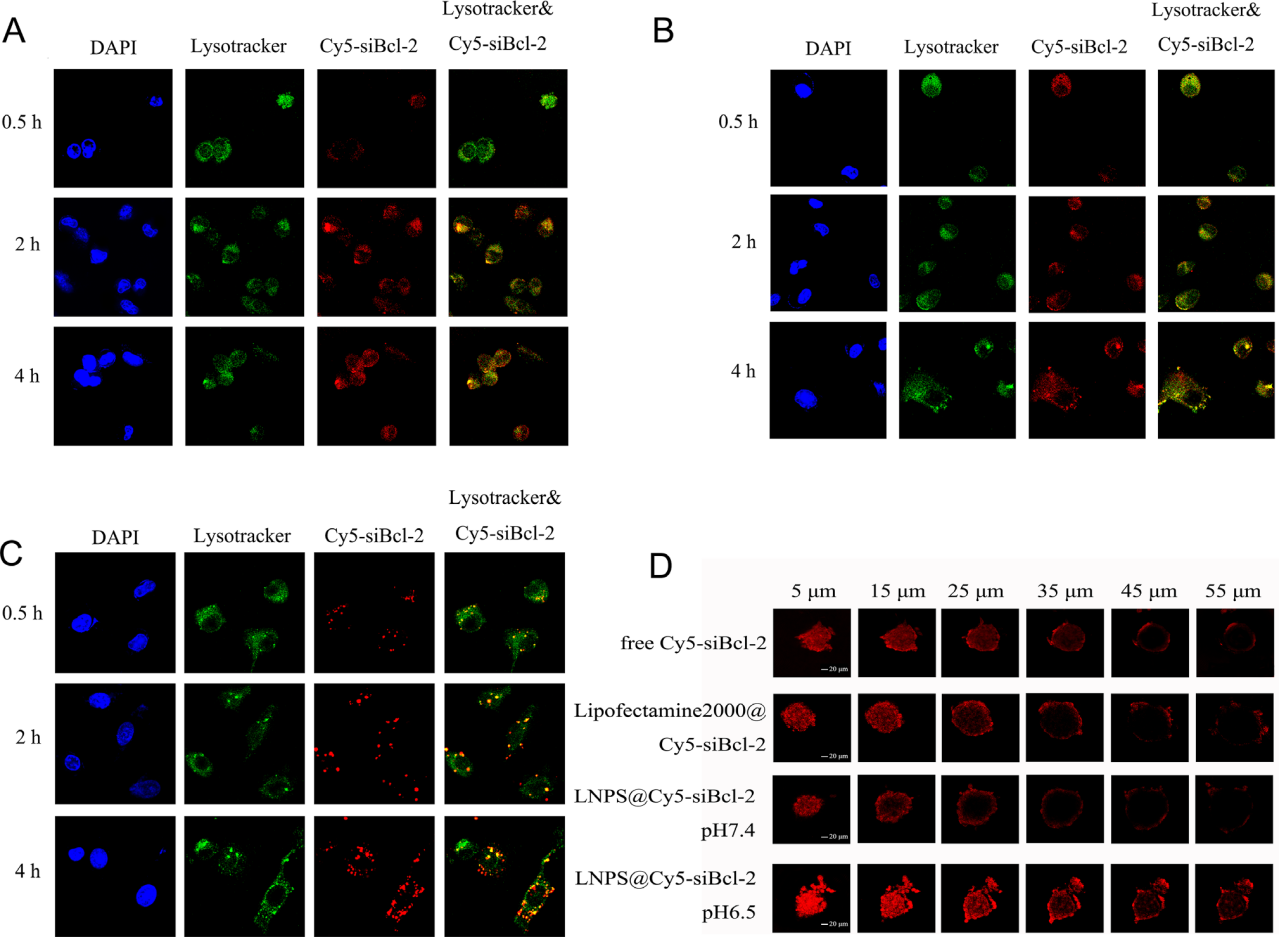
Intracellular trafficking of Cy5-siBcl-2 in A549 cells and the penetration of Cy5-siBcl-2 in tumor spheroid of A549 cells delivered by LNPS@Cy5-siBcl-2 (Panel **A** and panel **B**) are the subcellular distribution of Cy5-siBcl-2 delivered by LNPS@Cy5-siBcl-2 in pH7.4 and pH 6.5 medium, respectively. (Panel **C**) is the subcellular distribution of Cy5-siBcl-2 delivered by Lipofectamine2000@Cy5-siBcl-2 in pH7.4 medium. (Panel **D**) is the penetration of Cy5-siBcl-2 in tumor spheroid of A549 cells. 20 × oil immersion objective and 10 × ocular lens. Red stands for Cy5-siBcl-2, green stands for endolysosome and blue stands for nucleus. Yellow stands for Cy5-siBcl-2 in endolysosome.

The intracellular trafficking of Cy5-siBcl-2 delivered by LNPS@Cy5-siBcl-2 in A549 cells was observed by CLMS, and the results are shown in Figure 7A–7C. When LNPS@Cy5-siBcl-2 and Lipofectamine2000@Cy5-siBcl-2 were incubated with A549 cells in 2 h, lots of Cy5-siBcl-2 localized in the lysosome. When they were incubated with A549 cells for 4 h, much more Cy5-siBcl-2 escaped from lysosome, and the red fluorescence of Cy5-siBcl-2 out of lysosome significantly increased. Furthermore, as shown in Figure 7C, the red fluorescence was agglomerate distribution in cytoplasm when Lipofectamine2000@Cy5-siBcl-2 was incubated with A549 cells, which indicated that Cy5-siBcl-2was aggregated with lipofectamine2000 in cytoplasm, consequently resulting in the low transfection activity. However, as shown in Figure 7A–7B, the red fluorescence punctate distribution in cytoplasm when LNPS@Cy5-siBcl-2 was incubated with A549 cells, which indicated that Cy5-siBcl-2 released from calcium phosphate core in cytoplasm, and consequently resulting in the high transfection activity.

### The penetration of Cy5-siBcl-2 in tumor spheroid of A549 cells delivered by LNPS@Cy5-siBcl-2

The penetration of Cy5-siBcl-2 in tumor spheroid of A549 cells delivered by LNPS@Cy5-siBcl-2 was investigated by CLMS, and the results are shown in Figure 7D. LNPS@Cy5-siBcl-2 delivered much more Cy5-siBcl-2 into the deeper region of tumor spheroid in pH 6.5 medium as compared with what was in pH 7.4 medium.

### *In vivo* antitumor activity of LNPS@Cy5-siBcl-2

**Figure 8:**
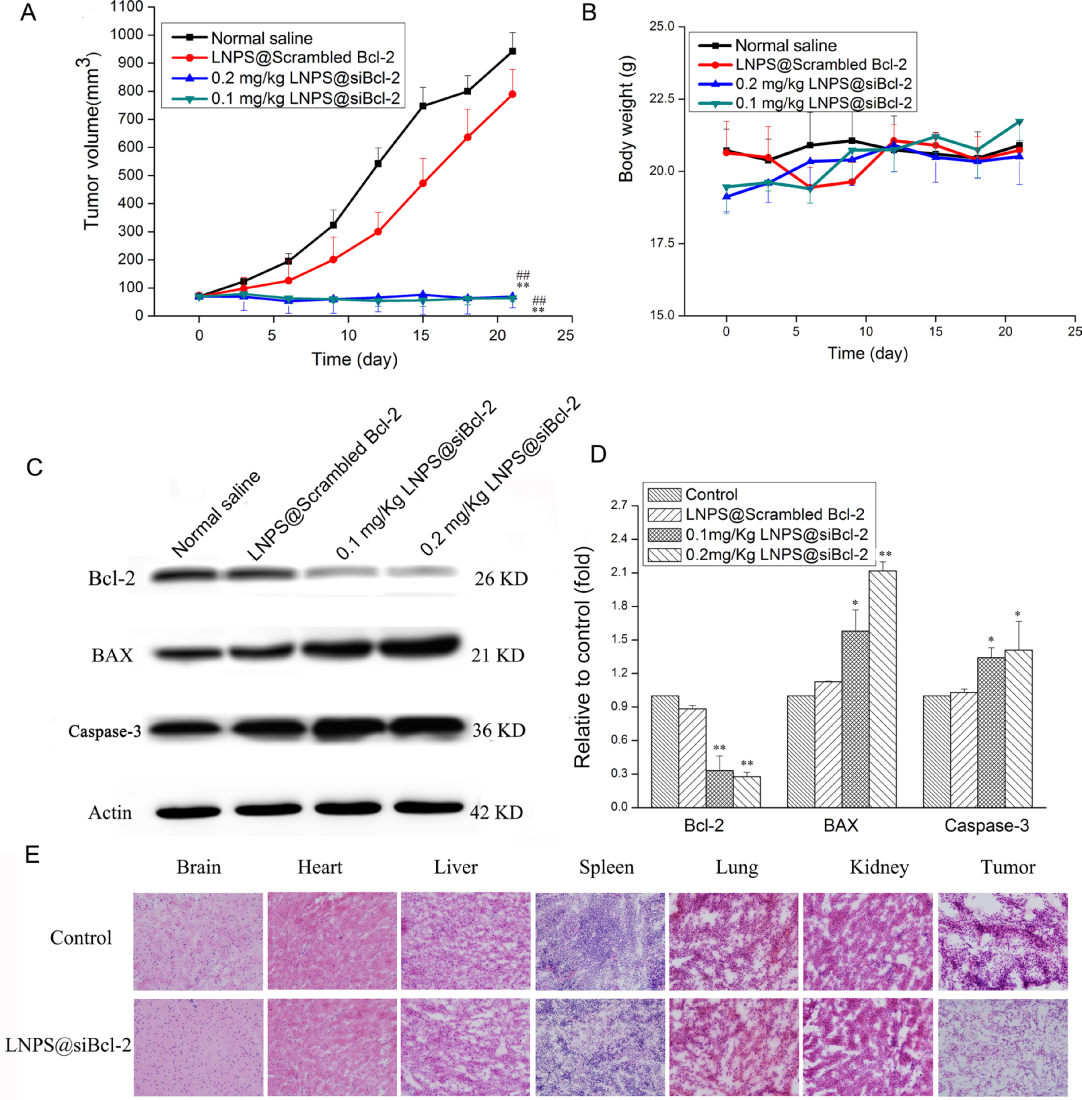
The *in vivo* antitumor activity of LNPS@siBcl-2 on tumor-bearing nude mice (Panel **A**) is the change in tumor volume. (Panel **B**) is the body weight change during treatment. (Panel **C**) is the Bcl-2, BAX and Caspase-3 expression in tumor tissue after tumor-bearing mice were treated with LNPS@Cy5-siBcl-2. (Panel **D**) is the statistic result of panel C. (Panel **E**) is representative H&E staining picture of major organs and tumor tissue. Data are expressed as the mean ± SD, *n* = 5. **P < 0.01, vs Normal Saline. ^##^*P* < 0.01, *vs* LNPS@Scrambled Bcl-2.

As shown in Figure 8A–8B, LNPS@Scrambled Bcl-2 could not inhibit tumor growth as compared with normal saline treated tumor-bearing mice. However, tumor growth was dramatically hindered by LNPS@siBcl-2 as compared with LNPS@Scrambled Bcl-2. In addition, LNPS@siBcl-2 did not cause significant body weight loss.

The protein expression of Bcl-2, BAX and Caspase-3 in tumor tissue was analyzed by Western Blot analysis, and the results are shown in Figure 8C–8D. Compared with normal saline and LNPS@Scrambled Bcl-2 treated tumor-bearing mice, the expression of Bcl-2 significantly reduced, and the expression of BAX and Caspase-3 obviously increased in tumor tissue after tumor-bearing mice were treated with LNPS@siBcl-2. Besides, there were no significant changes in Bcl-2, BAX and Caspase-3 expression in tumor tissue from LNPS@Scrambled Bcl-2 treated tumor-bearing mice as compared with what were from normal saline treated tumor-bearing mice.

The morphological changes of major organs and tumor tissue were observed by H&E staining, and the results are shown in Figure 8E. Compared with normal saline treated tumor-bearing mice, there were not obviously histopathological changes in brain, heart, liver, kidney, spleen, and lung after tumor-bearing mice were treated with LNPS@siBcl-2. However, compared with tumor tissue section from normal saline treated tumor-bearing mice, tumor tissue section from LNPS@siBcl-2 treated tumor-bearing mice showed the less viable tumor cells, notable necrosis and serious inflammatory cell infiltration. This indicated that LNPS@siBcl-2 was safe to normal organs but exhibited specific toxicity to tumor tissue.

### Biodistribution of Cy5-siBcl-2

**Figure 9:**
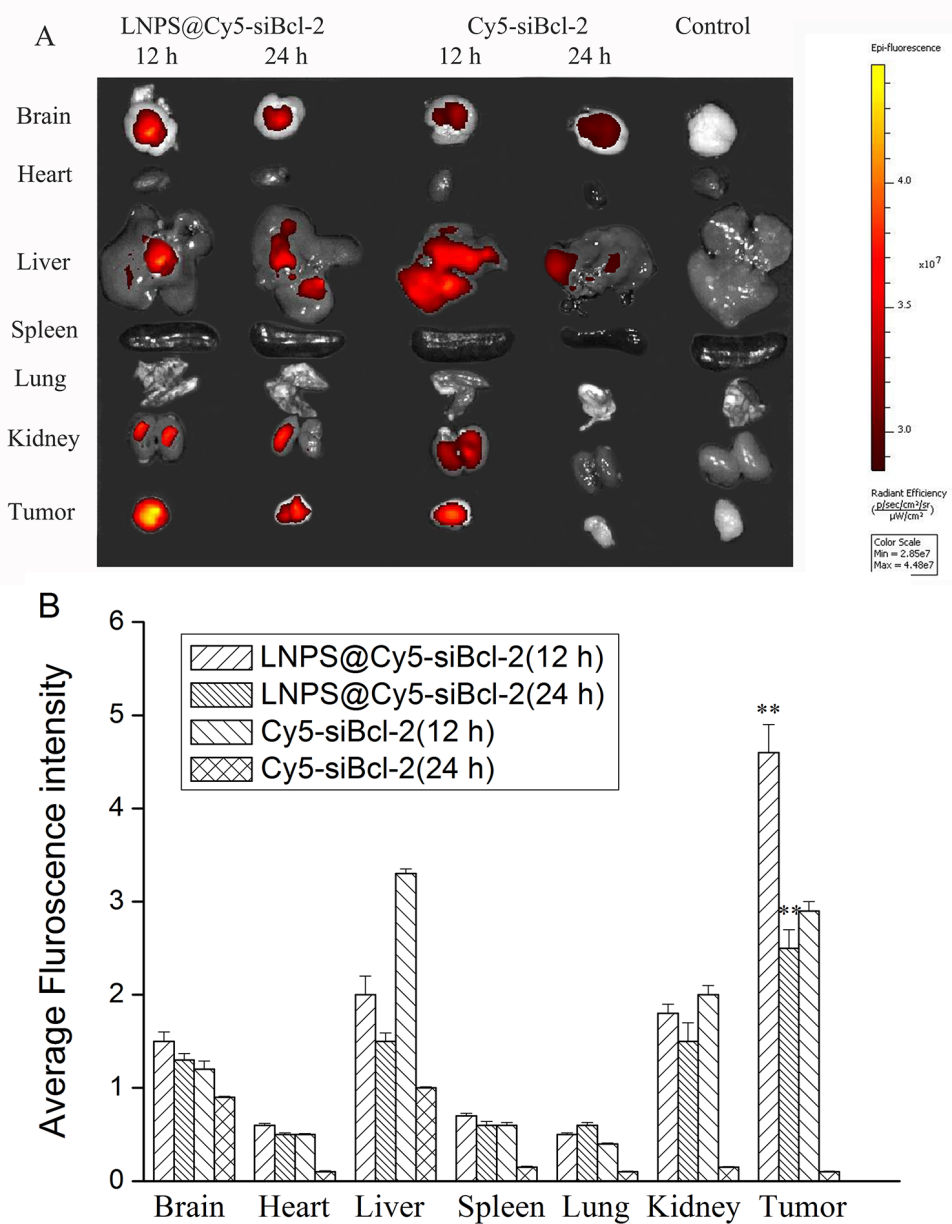
Biodistribution of Cy5-siBcl-2 in organs and tumor tissue after LNPS@Cy5-siBcl-2 or Cy5-siBcl-2 was administered to tumor-bearing mice by tail vein injection (**A**). Panel **B** is the statistic result of panel A. Data are expressed as the mean ± SD, *n* = 5. **P < 0.01, *vs* free Cy5-siBcl-2 at the same time point.

As shown in Figure 9, Cy5-siBcl-2 was mainly distributed in tumor tissue after the administration of LNPS@Cy5-siBcl-2 via tail vein injection. However, Cy5-siBcl-2 was distributed almost through the all organs after the naked Cy5-siBcl-2 was administered via tail vein injection. Besides, Cy5-siBcl-2 was still observed in tumor tissue at 24 h after LNPS@Cy5-siBcl-2 was intravenously injected into mice, but there was no Cy5-siBcl-2 red fluorescence in tumor tissue at 24 h after naked Cy5-siBcl-2 was injected into mice by tail vein.

**Figure 10:**
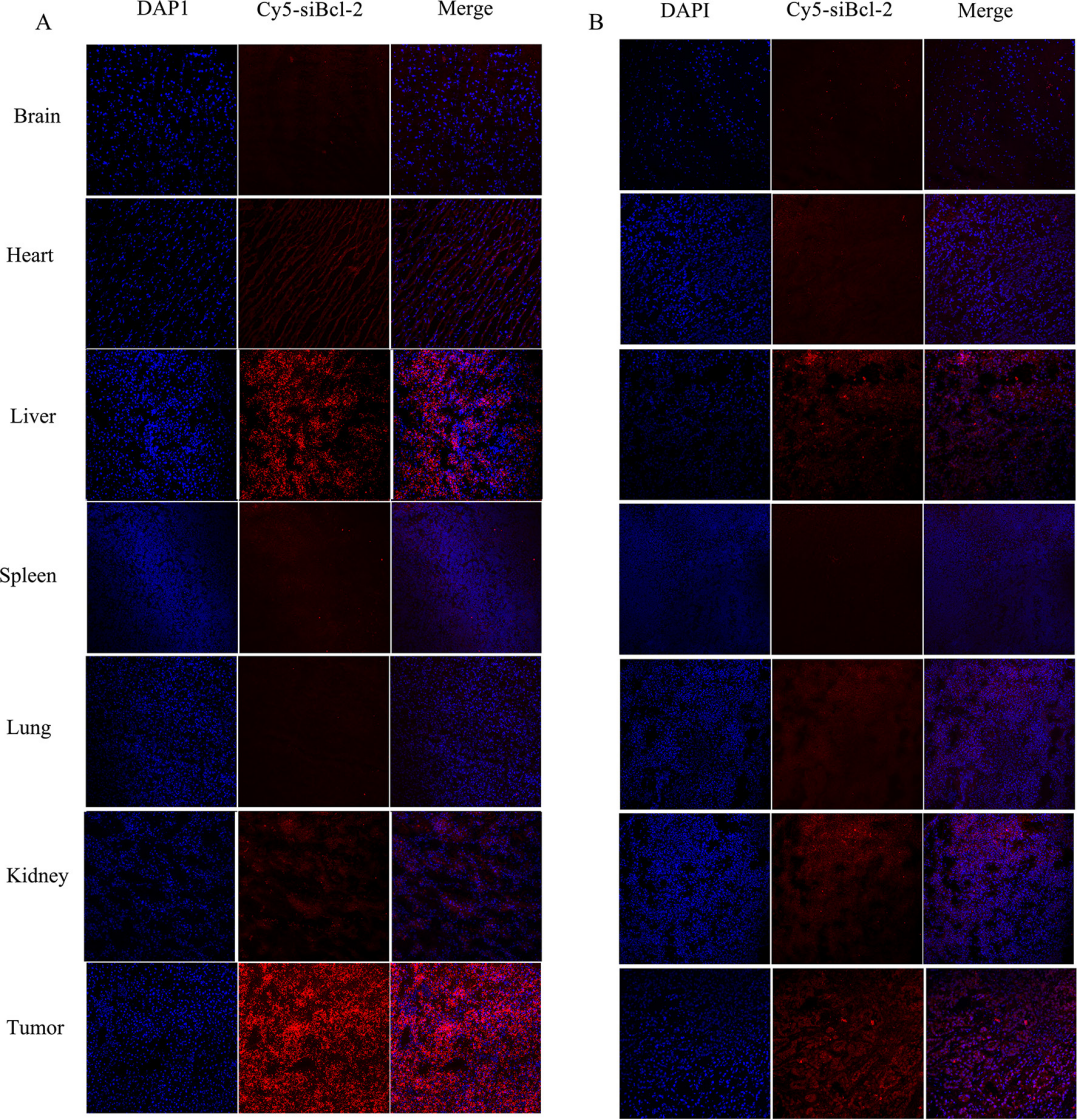
The distribution of Cy5-siBcl-2 in section of organs and tumor tissues from tumor-bearing mice at 12 h after LNPS@Cy5-siBcl-2 (panel **A**) or naked Cy5-siBcl-2 (panel **B**) was administered by tail vein injection.

The distribution of Cy5-siBcl-2 in the frozen section of tumor tissue and organs was observed by laser confocal scan microscopy, and the results are shown in Figure 10. A large amount of Cy5-siBcl-2 red fluorescence was extensively distributed in the section of tumor tissue from tumor-bearing mice treated with LNPS@Cy5-siBcl-2. After tumor-bearing mice were treated with naked Cy5-siBcl-2, a little amount of Cy5-siBcl-2 red fluorescence was distributed in the section of tumor tissue. The above results were consistent with the results of *in-vivo* image experiment.

## DISCUSSION

The development of non-toxic carrier is one of the major strategies for the RNAi based therapy. Calcium phosphate has been widely used in gene transfection on cells because of its bigger specific surface area and higher capacity to deliver a broad variety of agents. Up to now, calcium phosphate nanoparticles are mainly classified into lipid coated and polymer coated particles. However, the aggregation of calcium phosphate particles leads to its poor colloidal stability, which limited its clinic application [26]. Thus, it is of great significance to increase the transfection efficiency and colloidal stability of calcium phosphate nanoparticles.

There were two steps to prepare LNPS@siBcl-2. Firstly, the reverse micro-emulsion method was applied to forming CaP core. Secondly, the thin film disperse method was used to prepare the lipid hybrid nanoparticles. The DOPA, an anionic lipid, was used to coat and stabilize the siBcl-2 loaded CaP core. A cationic lipid DOTAP was used as the outer leaflet lipid to form an asymmetric lipid bilayer structure [26, 35]. In this paper, in order to improve the transfection activity of siBcl-2 *in vivo*, a pH triggered charge reversible material CHOL-AA-Cit was applied to creating the charge reversible calcium phosphate lipid hybrid nanoparticle.

The process of charge reversion was usually traced by the reversal of zeta potential [27] Thus, the change of zeta potential and particle size of nanoparticle were detected to confirm the micro environmental pH triggered charge reversal property. The results indicated that the zeta potential and particle size of LNPS@siBcl-2 were pH sensitive. The size of LNPS@siBcl-2 in acidic tumor microenvironment became bigger, which resulted from the aggregation and disassembly of nanoparticles in acidic environment. The disassembly of nanoparticles in acidic environment was probably due to: (1) CaP core dissolved and became loose in acidic environment; (2) the degradation of CHOL-AA-Cit in acidic environment led to the exposure of positively charged amines, which resulted in the enhancement of the interaction between positively charged conjugate and cationic DOTAP, subsequently decreasing the stability of outer leaflet lipid. Consequently, the stability of LNPS@siBcl-2 was decreased in acidic microenvironment.

The escape of siRNA from endolysosome is of great significance for its transfection activity since siRNA can be degraded in the endolysosome [28]. One aim of this paper was to enhance the siRNA escape from the endolysosome. The endolysosome escape capacity of nanoparticle was evaluated by hemolytic experiments *in vitro* by using rat blood red cell. According to the American Society for Testing and Materials (ASTM F756-00, 2000), a material was classified as non-hemolytic (0~2% of hemolysis), slightly hemolytic (2~5% of hemolysis), and hemolytic (> 5% of hemolysis) [29]. As shown in Table 1, the results of hemolytic experiment indicated that the LNPS@siBcl-2 was classified as non-hemolytic in blood circulation and hemolytic at pH5.0. These results implied that LNPS@siBcl-2 could not cause hemolysis in blood, but it could damage the endolysosome membrane at pH5.0, which could enhance the escape of siBcl-2 from endolysosome, subsequently increasing the transfection activity of LNPS@siBcl-2. Thus, compared with Lipofectamine2000@siBcl-2, the transfection activity and cytotoxicity of LNPS@siBcl-2 on A549 cells was greater at the same concentration of siBcl-2 (Figure 3).

The results of subcellular distribution of Cy5-siBcl-2 in A549 cells showed that LNPS@Cy5-siBcl-2 could escape from the endolysosome and release the Cy5-siBcl-2 into cytoplasm (Figure 7). These results were well consistent with hypothesis that the integration of CHOL-AA-Cit into the lipid hybrid calcium phosphate nanoparticles intensely facilitates the endolysosomal escape of the nanoparticle. One reason was that LNPS@Cy5-siBcl-2 became positively charged in endolysosome, which increased the interaction between nanoparticle and endolysosomal membrane. The result of the hemolysis experiment was a strong evidence for this hypothesis. Another reason was that a little of CaP was dissolved at acidic endolysosome. Subsequently, the dissolved calcium and phosphate ions increased the osmotic pressure and resulted in the swelling of endolysosome, consequently enhancing the escape of siRNA [30].

To highlight the functions of CHOL-AA-Cit in lipid hybrid calcium phosphate nanoparticle, the lipid hybrid calcium phosphate nanoparticles without CHOL-AA-Cit (NLNPS@Cy5-siBcl-2) were prepared. The intracellular trafficking of Cy5-siBcl-2 delivered by NLNPS@Cy5-siBcl-2 is shown in Supplementary Figure 4. There were differences in endolysosome escape for two formulations. A549 cells treated with NLNPS@Cy5-siBcl-2 displayed more yellow region in the cell, which indicated Cy5-siBcl-2 localized in the endolysosome. However, the cells treated with LNPS@Cy5-siBcl-2 showed much more single red region in the cell, which indicated Cy5-siBcl-2 localized out of endolysosome.

The cellular uptake mechanism of LNPS@siBcl-2 on A549 cell was investigated, the results indicated that the cellular uptake of LNPS@siBcl-2 on A549 cell was significantly reduced at the present of colchicine (Figure 6). This result indicated that the major pathway of endocytosis of LNPS@Cy5-siBcl-2 was micropinocytosis on A549 cells. It was reported that energy consumption was usually involved in the macropinocytosis process [31, 32]. The experiment results further indicated that the cellular uptake of LNPS@siBcl-2 could be inhibited by low temperature (4°C) and ATP depletion. These results implied that the cellular uptake of LNPS@Cy5-siBcl-2 was also an energy-consuming process.

Bcl-2 is an anti-apoptosis gene that can suppress cell apoptosis and protect cell from various cytotoxic attack. BAX is a pro-apoptosis gene that can promote cell apoptosis [33]. Based on the subcellular distribution of the Cy5-siBcl-2 delivered by calcium phosphate lipid hybrid nanoparticles, the LNPS@siBcl-2 was expected to be effective in gene silencing effect at the cell level. The experimental results indicated that LNPS@siBcl-2 could significantly decrease the expression of Bcl-2 and increase the expression of BAX and Caspase-3 in A549 cells (Figure 3), which consequently resulted in the high cytotoxicity on A549 cells.

The *in vivo* antitumor activity of LNPS@siBcl-2 was performed in tumor xenograft model in female nude mice. The tumor growth was significantly suppressed by LNPS@siBcl-2. To demonstrate the mechanism of antitumor activity of LNPS@siBcl-2 *in vivo*, tumor tissue was excised at 24 h after the last treatment of LNPS@Cy5-siBcl-2. The protein expression level of Bcl-2, BAX and Caspase-3 in tumor tissue was analyzed by Western Blot. The results demonstrated that inhibition of tumor growth was related with the decrease of Bcl-2 protein expression and the increase of the BAX and Caspase-3 protein expression (Figure 8). In addition, the biodistribution of Cy5-siBcl-2 delivered by LNPS@Cy5-siBcl-2 in tumor-bearing nude mice implied that LNPS@Cy5-siBcl-2 could protect Cy5-siBcl-2 from degradation and kept longer time in the bloodstream, which led to significant accumulation of Cy5-siBcl-2 in tumor tissue (Figure 9 and Figure 10). In a word, the passive tumor targeting property and high transfection activity of LNPS@siBcl-2 resulted in its great antitumor activity *in vivo*.

## MATERIALS AND METHODS

### Materials

1,2-dioleoyl-3-trimethylammonium-propanechloridesalt (DOTAP), 1,2-dioleoyl-sn-glycero-3-phosphate (DOPA) were purchased from Avanti Polar Lipids, Inc. (Alabaster, AL). Cholesterol (CHOL), aminocaproicacid (AA), calcium chloride (CaCl_2_), dibasic sodium phosphate, dicyclohexylcarbodiimide (DCC), and dimethylaminopyridine (DMAP) were obtained from J&K CHEMICA (Beijing, China). Citraconic anhydride (Cit) and cyclohexane/Igepal CO-520 were purchased from Sigma-Aldrich Company (St. Louis, MO, USA). Lipofectmine2000, 4′,6-diamidino-2-phenylindole (DAPI), (3-(4,5-dimethylthiazolyl-2)-2,5-diphenyltetrazolium bromide) (MTT) and Lysotracker Green were purchased from Invitrogen Technologies Company (Carlsbad, USA). Anti-Bcl-2 antibody was purchased from Abcam (Cambridge Science Park, British). Anti-β-actin antibody, anti-BAX antibody, anti-Caspase-3 and anti-rabbit IgG H&L (HRP) were purchased from Proteintech (Chicago, USA).

siBcl-2 was purchased from Guang Zhou Ribobio Co.,Ltd. (Guangzhou, Guangdong Province, China), and the sequences were as follows: target sense, GGATGACTGAGTACCTGAA. Sense strand, 5′GGAUGACUGAGUACCUGAA dTdT 3′. Antisense strand, 3′ dTdT CCUACUGACUCAUGGACUU5′.

A549 cell line was purchased from Institute of Biochemistry and Cell Biology, Chinese Academy of Science, Shanghai, China. Cells were cultured in DMEM with 10% fetal bovine serum, 1% mixture solution of penicillin and streptomycin. Cells were placed in 5% CO_2_ at 37°C under fully humidified conditions for proliferation, and culture media was refreshed every 2 days.

The 6-week-old BALB/c nude mice (the body weight was about 21~22 g) were purchased from Experimental Animal Center of Fourth Military Medical University. Animal experiment was carried out in accordance with the protocols that were approved by the Animal Care and Use Committee, Fourth Military Medical University (approval number: 16010).

### Synthesis of pH-sensitive conjugate CHOL-AA-Cit

The synthetic route of CHOL-AA-Cit is shown in Supplementary Figure 5. Cholesterol-aminocaproicacid conjugate (CHOL-AA) was synthesized by the reported procedure [34]. To prepare cholesterol-aminocaproicacid-citraconic conjugate (CHOL-AA-Cit), CHOL-AA (500 mg, 1 mmol) was dissolved in 10 mL anhydrate dichloromethane and several drops of trimethylamine were added. Then the citraconic anhydride (169 mg, 1.5 mmol) was added to the reaction mixture. Subsequently, the solution was stirred for 2 h at 0°C. The process was monitored by TLC. The reaction mixture was washed with saturated sodium chloride solution for three times, and the organic phase was dried under sodium sulfate. After the removal of the sodium sulfate by filtering, the organic solvent was evaporated, and the residue was purified by flash chromatography on silica gel to get product as a faint yellow powder. The product was confirmed by ^1^H NMR and MS analysis.

### Preparation and characterization of LNPS@siBcl-2

A water-in-oil micro-emulsion method was applied to preparing the calcium phosphate nanoparticle [35]. In simple terms, 150 μL of 500 mM CaCl_2_ and 60 μL of 1.4 mg/mL siBcl-2 were incubated for 15 minutes at room temperature. The above mixture was dispersed in 5 mL cyclohexane/Igepal CO-520 (75/25, V/V) solution under stirring to form calcium phase. The 150 μL of 25 mM Na_2_HPO_4_ was dispersed in another 5 mL cyclohexane/Igepal CO-520 (75/25, V/V) solution to get phosphate phase by the same condition. Then 150 μL (20 mg/mL) dioleoylphosphatydicacid (DOPA) in chloroform was added. After that, the calcium phase was added dropwise into the phosphate phase. Then the mixture was stirred for 30 min at the room temperature. Then, 15 mL of absolute ethanol was added to the mixture to generate the calcium phosphate nanoparticles. The solution was stirred for 30 min. The precipitate was centrifuged at 12,000 rpm/min for 30 min at 4°C to remove cyclohexane and surfactant. The precipitate was washed with absolute ethanol for two times and placed in a vacuum drier.

The thin film hydration method was applied to preparing the siBcl-2-loaded calcium phosphate lipid hybrid nanoparticles (LNPS@siBcl-2). Briefly, CaP core was dissolved with chloroform containing DOTAP/CHOL-AA/CHOL-AA-Cit. According to the diameter and zeta potential of the nanoparticle in pH7.4 medium and in pH5.0 medium, the ratio of DOTAP/CHOL-AA/CHOL-AA-Cit was optimized. After evaporating chloroform, the lipid film was dispersed in 6 mL deionized water to form LNPS@siBcl-2. Then the LNPS@siBcl-2 solution was extruded through a 220 nm polycarbonate membrane. The dynamic light scattering (Beckman Coulter Particle Analyzer, Fullerton, California, USA) was used to detect zeta potential and particle size of LNPS@siBcl-2. All experiments were done in triplicate. The morphology of LNPS@siBcl-2 was observed by transmission electron microscopy (TEM, JEOL-100CXII, Japan) [36]. siBcl-2 loaded calcium phosphate lipid hybrid nanoparticles without CHOL-AA-Cit (NLNPS@siBcl-2) were prepared by using the same method.

### Charge reversible property

After LNPS@siBcl-2 was dispersed in different PBS (pH7.4 and pH5.0), the zeta potential and size were detected by dynamic light scattering.

The hemolysis rate of LNPS@siBcl-2 was evaluated by using UV-vis spectrophotometry test. Fresh blood samples were obtained from rat and were anticoagulated with sodium heparin. Then red blood cells were collected and washed with normal saline for three times. The red cells were diluted with isotonic PBS buffer (pH7.4 and pH5.0) [37, 38]. Different concentration of LNPS@siBcl-2 was incubated with red cell suspension at 37°C. The PBS buffer and deionized water were used as negative control and positive control, respectively. After 4 h, red cell suspension was centrifuged, and the absorbance of the supernatant at 414 nm was detected by UV-vis spectrophotometry.

### Gene silencing effect of LNPS@siBcl-2 on A549 cells

For gene silencing experiments, A549 cells were seeded in 6-well plates one day before transfection. A549 cells were treated with LNPS@siBcl-2 for 12 h (the concentration of siBcl-2 was 10, 15 and 25 nmol/L). After that, cells were lyzed with RIPA lysis buffer. The cells lysis was centrifugated and the supernatant was collected. The protein concentration in the supernatant was quantified by Bradford reagent. The expression of Bcl-2, Caspase-3 and BAX was detected by Western Blot [39].

The cytotoxicity of calcium phosphate lipid hybrid nanoparticles without loading siBcl-2 (LNPS), LNPS@siBcl-2 and NLNPS@siBcl-2 was detected on A549 cells by MTT assay [40]. A549 cells were seeded in 96-well plate and cultured for 24 h. The media was replaced by fresh one that contained different concentration of LNPS, LNPS@siBcl-2 and NLNPS@siBcl-2. After incubation for another 48 h, the MTT solution (5 mg/mL) was added to the well of plate and incubated for 4 h. Finally, after the removal of culture medium, 200 μL DMSO was added to the well and its absorbance at 490 nm was detected by microplate reader (Bio-Rad Laboratories, Inc. Richmond, California, USA). The scrambled Bcl-2 loaded calcium phosphate lipid hybrid nanoparticle (LNPS@scrambled Bcl-2) was used as a negative control, and Lipofectamine2000@siBcl-2 was used as a positive control. The concentration of siBcl-2 was 1, 5, 10, 25 and 50 nmol/L.

### The cellular uptake of LNPS@Cy5-siBcl-2

A549 cells were seeded in 6-well plate and incubated for 24 h. Then the cells were incubated with LNPS@Cy5-siBcl-2 at the concentration of 25 nmol/L (Cy5-siBcl-2). The medium pH was 7.4 or 6.5. After being incubated for 0.5, 2 and 4 h, the culture medium was removed and the cells were slightly rinsed with PBS. The cells were collected and re-suspended in 500 μL PBS. Finally, the cells were subject to flow cytometric analysis by using flow cytometry (Becton Dickinson FACS can, USA). The Lipofectamine2000@Cy5-siBcl-2 was used as positive control.

To study the cellular uptake mechanism of LNPS@Cy5-siBcl-2, A549 cells were placed in 4°C environment for 1 h or pre-incubated with ATP depletion reagent (mixture of 10 mmol/L sodium azide and 6.5 mmol/L 2-deoxidation glucose) at 37°C for 1 h. After that, LNPS@Cy5-siBcl-2 was added to the culture medium and incubated for 4 h at 4°C or 37°C. A549 cells were collected and detected by flow cytometry. In addition, A549 cells were pre-incubated with different endocytic inhibitors such as sucrose, methyl-β-cyclodextrin and colchicine for 1 h [41]. After that, LNPS@Cy5-siBcl-2 was added to the culture medium and incubated for 4 h at 37°C. A549 cells were collected and detected by flow cytometry.

The cellular uptake of LNPS@Cy5-siBcl-2 and NLNPS@siBcl-2 on A549 cells was also detected by laser confocal scan microscopy. Briefly, A549 cells were seeded in 24-well plate with a cover glass and incubated overnight. The medium was replaced with fresh culture medium containing LNPS@Cy5-siBcl-2 at concentration of 25 nmol/L (Cy5-siBcl-2). The medium pH was 7.4 or 6.5. After being incubated for 0.5, 2 and 4 h, A549 cells were rinsed three times by PBS. Then the cells were stained with DAPI for 15 min. After that, the cells were washed with PBS and fixed by 4% paraformaldehyde solution. The glass was placed on the slide and observed by laser confocal scan microscopy (TCS SP2 confocal microscope,Leica, Germany) [42]. Meanwhile, the arbitrary fluorescence of Cy5-siBcl-2 was calculated by Image Pro Plus software.

### The intracellular trafficking of LNPS@Cy5-siBcl-2

The intracellular trafficking of LNPS@Cy5-siBcl-2 was detected by laser confocal scan microscopy. Briefly, A549 cells were seeded in 24-well plate with a cover glass and incubated overnight. The medium was replaced with fresh culture medium containing LNPS@Cy5-siBcl-2 (or Lipofectamine2000@Cy5-siBcl-2, or NLNPS@Cy5-siBcl-2) at concentration of 25 nmol/L (Cy5-siBcl-2). The medium pH was 7.4 or 6.5. After being incubated for 0.5, 2 and 4 h, A549 cells were rinsed three times by PBS. Then the cells were stained by using LysoTracker Green for 15 min. Then cells were stained with DAPI for 15 min. Finally, the cells were washed with PBS and fixed by 4% paraformaldehyde solution. The glass was placed on the slide and observed by laser confocal scan microscopy (TCS SP2 confocal microscope, Leica, Germany) [42].

### The penetration of Cy5-siBcl-2 in three-dimensional tumor spheroids

A549 cells were seeded at a density of 1 × 10^4^ cells per well in 24 plates coated by 300 μL of 2% low melting point agarose and were cultured for 15 days [43, 44]. to form three-dimensional tumor spheroids. Tumor spheroids were treated with LNPS@Cy5-siBcl-2, free Cy5-siBcl-2 and Lipofectamine2000@Cy5-siBcl-2 for 4 h at 37°C. The pH was 7.4 and 6.5 for LNPS@Cy5-siBcl-2 group. The concentration of Cy5-siBcl-2 was 50 nmol/L. Then the tumor spheroids were fixed with 4% paraformaldehyde for 10 min. Finally the spheroids were subject to laser confocal scan microscopy.

### *In vivo* antitumor activity of LNPS@siBcl-2

The xenograft tumor model was created by subcutaneous injection of A549 cells in the flank region of the nude mice. When the tumor volume was around 70 mm^3^, the 20 female nude mice were randomly divided into four groups. The mice were treated with normal saline, LNPS@Scrambled Bcl-2 (0.2 mg/kg) and LNPS@siBcl-2 (0.1 mg/kg and 0.2 mg/kg) by intravenous injection every three days for seven times. Tumor growth was monitored through measuring the perpendicular diameter of the tumor by using a caliper. The estimated volume was calculated according to the following formula: tumor volume (mm^3^) = 0.5 × length × width^2^. At the end of experiment, the Western Blot was applied to detecting the protein expression of Bcl-2, BAX and Caspase-3 in tumor tissue. Furthermore, the tumor tissues and organs were cut to 4 μm slices to perform H&E staining to observe histopathological changes.

### Distribution of Cy5-siBcl-2 in tumor-bearing nude mice

LNPS@Cy5-siBcl-2 and free Cy5-siBcl-2 were intravenously injected into the tumor-bearing nude mice by the tail vein. The dose of Cy5-siBcl-2 was 0.1 mg/kg. Mice were sacrificed at 12 h and 24 h after the tail vein injection. The brain, heart, liver, kidney, spleen, lung, and tumor were collected. The fluorescence intensity of Cy5-siBcl-2 in organs and tumor tissues was detected by the Caliper IVIS Lumina II *in-vivo* image system (Caliper Life Science, USA). At the same time, the frozen tumor tissues and organs were cut into 4 μm slices. Then the slices were stained with DAPI and fixed by 4% paraformaldehyde solution. After that, the Cy5-siBcl-2 fluorescence in the slices was observed by using laser confocal scan microscopy.

### Statistical analysis

Data were expressed as the mean±standard deviation. The comparison of each group was assessed by using one-way ANOVA analysis of SPSS software. The level of significance was indicated by *p < 0.05, **p < 0.01.

## CONCLUSIONS

The charge reversible calcium phosphate lipid hybrid nanoparticle decorated by CHOL-AA-Cit could effectively condense siBcl-2 and protect siBcl-2 from degradation. LNPS@siBcl-2 exhibited excellent transfection activity both *in vitro* and *in vivo*. LNPS@siBcl-2 has potential in the treatment of lung cancer.

## SUPPLEMENTARY FIGURES


