# Supplementary material for: Charge reversible calcium phosphate lipid hybrid nanoparticle for siRNA delivery
Source: Oncotarget. 2017 Apr 27;8(26):42772–88. doi: 10.18632/oncotarget.17484 (PMC5522105; doi:10.18632/oncotarget.17484)
Supplement: Supplementary file 1 [file oncotarget-08-42772-s001.pdf]

Charge reversible calcium phosphate lipid hybrid nanoparticle for siRNA delivery

Supplementary Materials

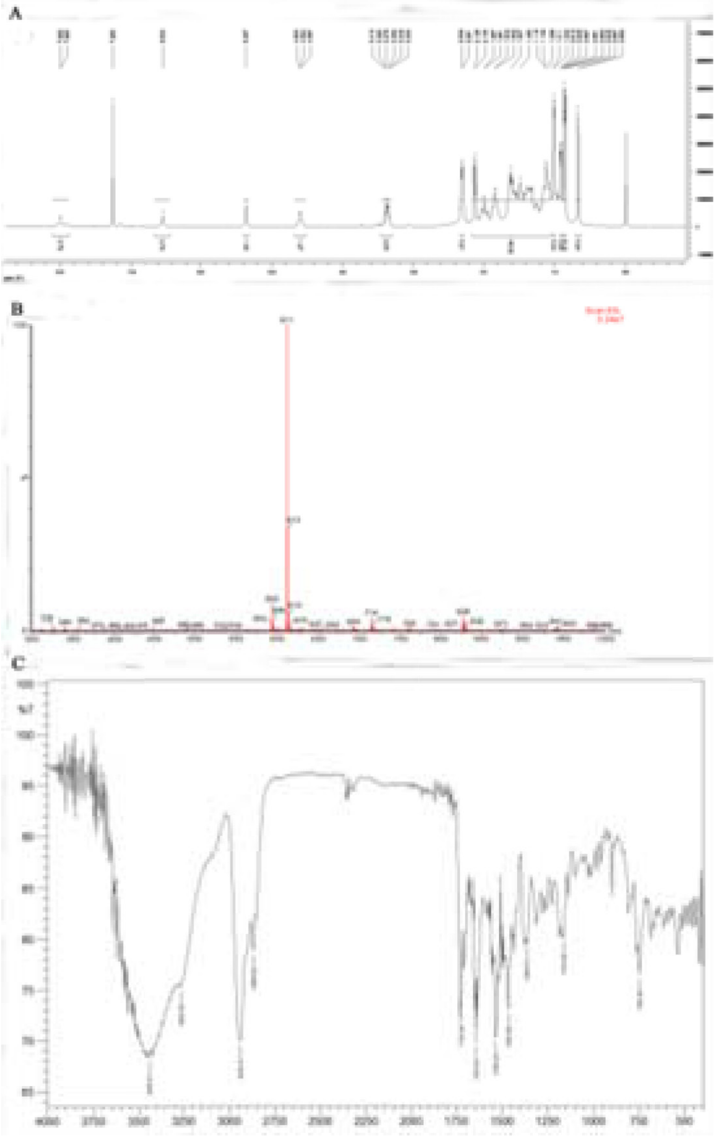

Supplementary Figure 1: The ^{1}H NMR spectrum, mass spectrum and FTIR spectrum of CHOL-AA-Cit.

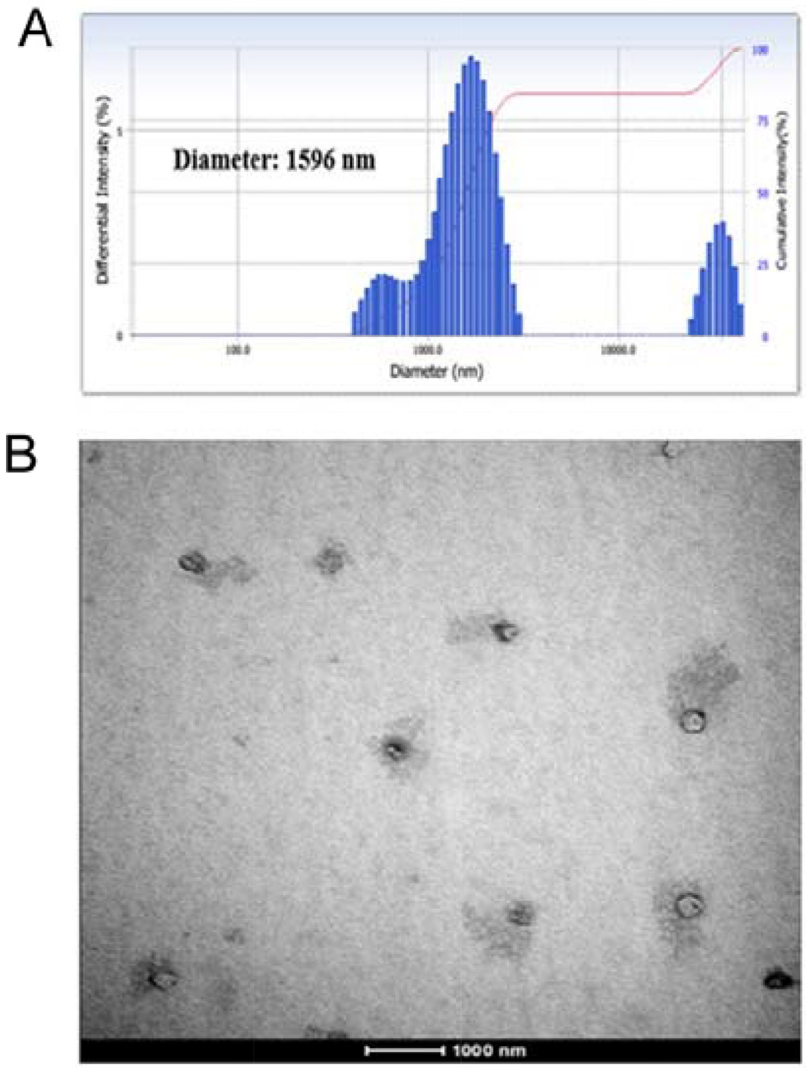

Supplementary Figure 2: The size of LNPS@siBcl-2 in pH5.5 medium detected by dynamic light scattering (**A**) and transmission electron microscopy (**B**).

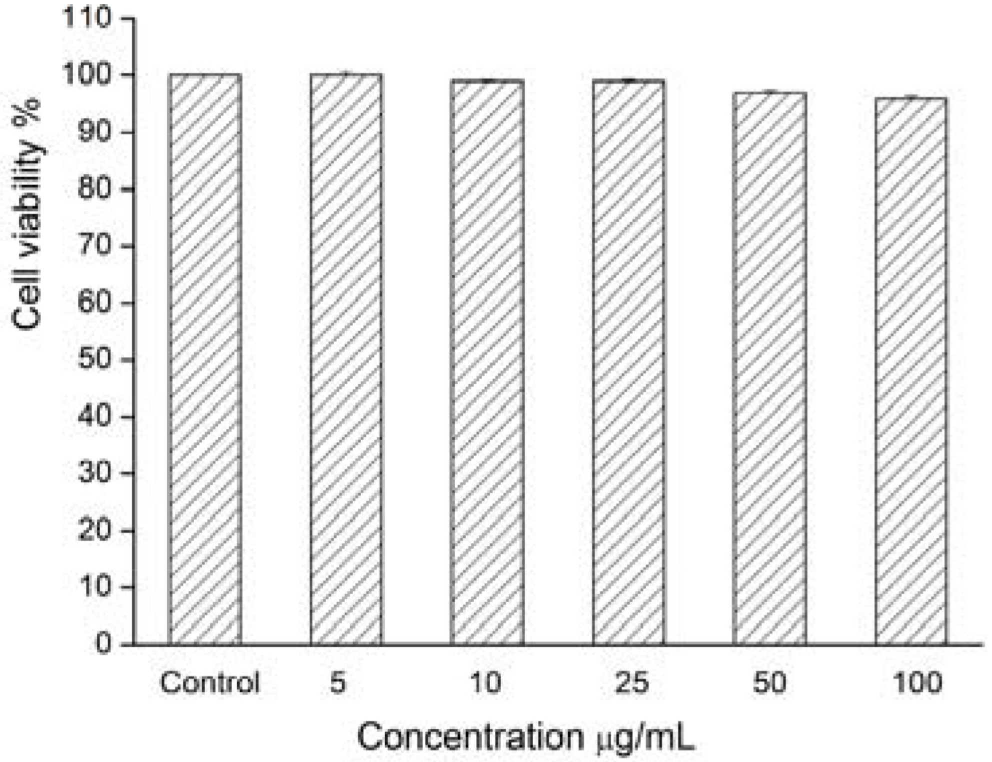

Supplementary Figure 3: Cytotoxicity of the calcium phosphate lipid hybrid nanoparticles without loading siBcl-2 (LNPS) on A549 cells.

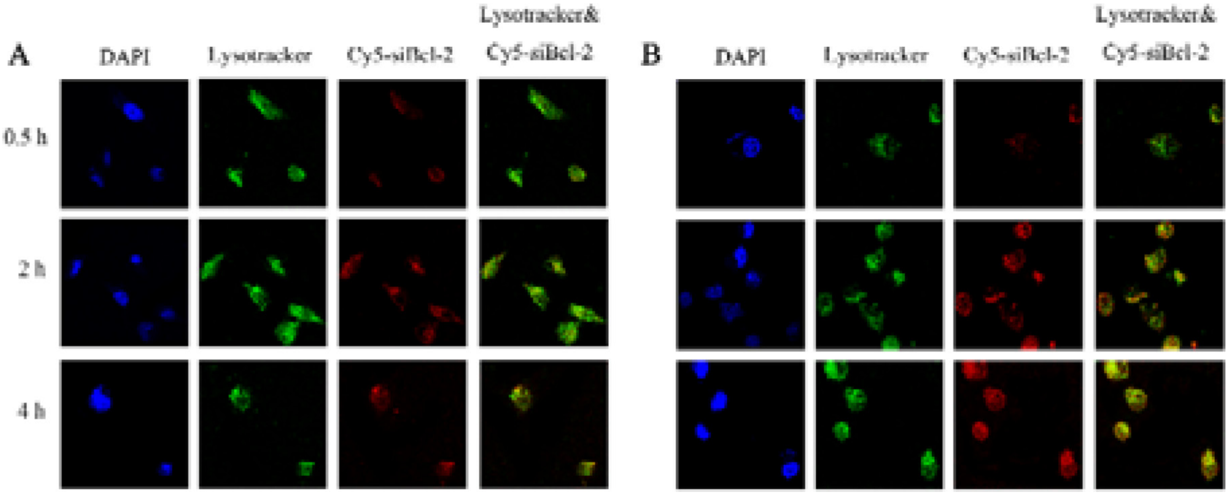

Supplementary Figure 4: Intracellular trafficking of Cy5-siBcl-2 in A549 cells delivered by NLNPS@Cy5-siBcl-2. (Panel **A** and panel **B**) are the subcellular distribution of Cy5-siBcl-2 delivered by NLNPS@Cy5-siBcl-2 in pH7.4 and pH 6.5 medium, respectively. 20 × oil immersion objective and 10 × ocular lens. Red stands for Cy5-siBcl-2, green stands for endolysosome and blue stands for nucleus. Yellow stands for Cy5-siBcl-2 in endolysosome.

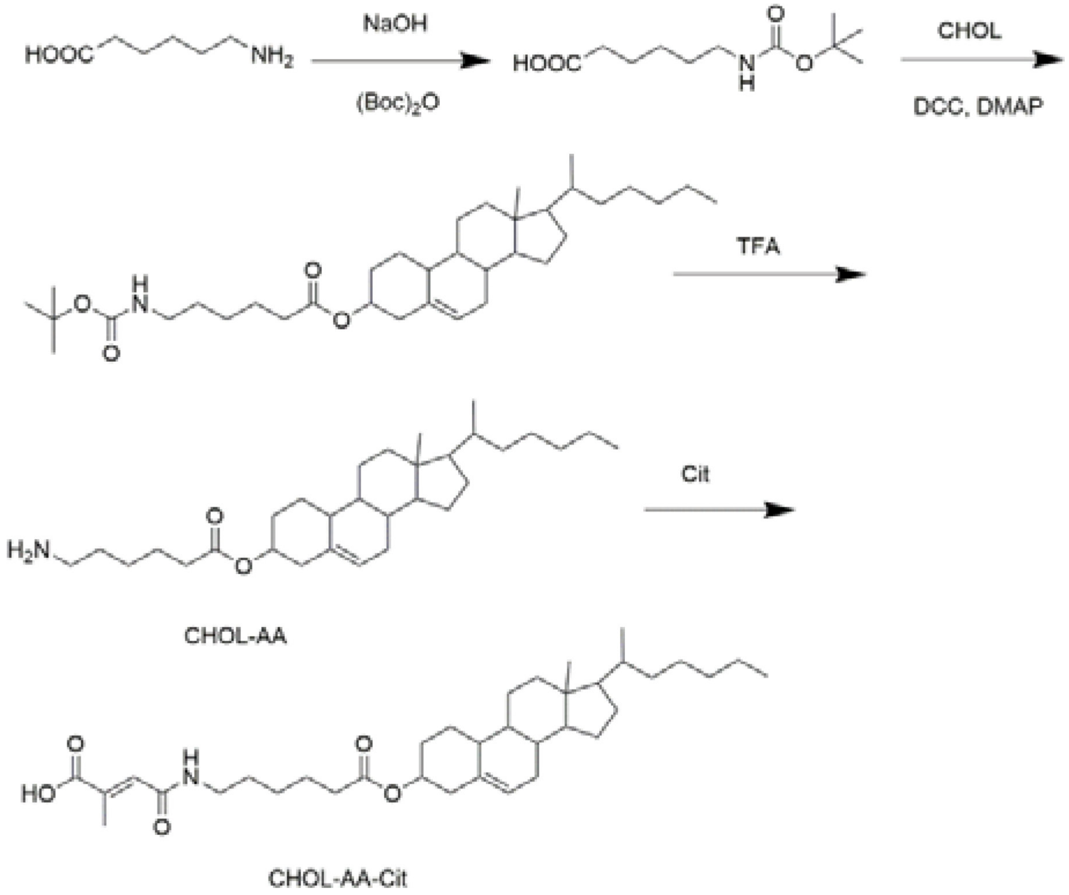

Supplementary Figure 5: Synthetic route of CHOL-AA-Cit.
